# The prevalence risk of anxiety and its associated factors among university students in Malaysia: a national cross-sectional study

**DOI:** 10.1186/s12889-021-10440-5

**Published:** 2021-03-04

**Authors:** Nurul Elyani Mohamad, Sherina Mohd Sidik, Mehrnoosh Akhtari-Zavare, Norsidawati Abdul Gani

**Affiliations:** 1 Department of Psychiatry, Faculty of Medicine & Health Sciences, Universiti Putra Malaysia, Serdang, Selangor Malaysia; 2 Cancer Resource & Education Center, Universiti Putra Malaysia, 43400 Serdang, Selangor Malaysia; 3 Department of Public Health, Faculty of Health, Tehran Medical Sciences, Islamic Azad University (IAU), Tehran, Iran

**Keywords:** Anxiety; university students, Malaysian, A national study

## Abstract

**Background:**

Anxiety disorder is one of the most common mental health problems worldwide, including Malaysia, and this issue has gained concern and attention from many, including experts and authorities globally. While average levels of stress and worry may help to motivate students to perform well in their studies, excessive feelings will increase their level of anxiety.

**Methods:**

A cross-sectional study was conducted at selected government and private universities throughout Malaysia. A total of 1851 students participated in this study. The students were asked to complete self-administered questionnaires, including socio-demographic, academic, and psychosocial characteristics. The Generalized Anxiety Disorder-7 (GAD-7) questionnaire was used to measure the prevalence risk of anxiety among the students. Chi-square analysis was conducted to find the relationship between the variables and anxiety, and multivariate logistic regression analysis was used to identify the predictors.

**Results:**

The response rate was 97.90%, where 1821 out of 1860 students participated in the study. The prevalence risk of anxiety in this study was recorded at 29%. The data revealed that academic year, financial support for the study, alcohol consumption, poor sleep quality, body mass index (BMI), having a good friend in the university, having doubt regarding the future, actively involved in the society, and having problems with other students and lecturer(s) were significantly associated with risk of anxiety; with the academic year as the primary predictor.

**Conclusions:**

The findings highlight the current prevalence risk of anxiety among university students in Malaysia. The outcome of this study can serve as the evident baseline data and help with the development of specific interventions in addressing and managing the issue appropriately.

## Background

Anxiety is a body’s normal response as a result of feeling worried, fearful, and stress about what is to come [1]. Anxiety can occur in anyone of any age. Occasional anxiety is common, but when it involves intense, persistent, and excessive fear and worry, it can exacerbate and lead to anxiety disorder [2, 3]. It can cause distress syndromes such as shaking, shortness of breath, headache, loss of mental power, anger, heart arrest, and many other syndromes [4]. The prevalence of anxiety in a general population was reported to be 3% [5]. Anxiety disorder varies from mild to severe cases and must be treated as it affects daily life. On the other hand, the prevalence of generalized anxiety disorder (GAD) in a general population was reported to be highest in a high-income country, and study stated that females, those who are below 60 years old, unmarried, have low educational levels, and small household income were statistically associated with GAD worldwide [6].

Located in Southeast Asia, Malaysia is a developing country that is undergoing rapid economic development. The prevalence of anxiety in South East Asia countries was reported to be between 2.1 to 5% [7]. According to a study conducted by Baxter et al. (2014), the prevalence of anxiety increases at the age of 10 to 19 years old and peaked at the age between 20 to 34 years old. Generally, university students are at an age where a high prevalence of anxiety was reported. Unlike high school, the university is not only academically challenging but also requires more attention in terms of social communication, homesickness, tuition fees, and cost of living, among other things [8]. This is justified by Shamsudin et al. (2014) which reported a higher rate of anxiety in students from public universities in Klang Valley, Malaysia [9]. With more than 590 higher education institutions; where 20 of them are government universities, education in Malaysia has always strived for better performance to achieve the targets and producing high-quality graduates to meet the current needs [10]. With the urge to improve their position in the QS World University Ranking, all universities have implemented different strategies to achieve the standard. Considering the disparate educational system nowadays, it can be a healthy growth, not only for the university but also for the country. However, the impact of this growth has been challenging, especially for the students to cope with the demands of tertiary education.

A high level of anxiety affects not only academic performance, but it can also cause many other detrimental effects such as depression, causing health to decline and suicide [11–14]. This study was conducted to identify the prevalence of anxiety and its associated factors among university students in Peninsular and East Malaysia. Determining the prevalence risk of anxiety and its correlation factors among university students are essential so that appropriate intervention programs can be implemented in this population.

## Methods

### Study design

A cross-sectional study was undertaken between June and December 2019 using self-administered questionnaires, including the GAD-7, to screen the students who had the risk of anxiety. The inclusion criteria for the students to participate in this study were Malaysian citizens, age 18 years and above, and are currently doing their tertiary education at the selected government or private universities in Malaysia. A total of 1860 undergraduate students were randomly selected to answer the questionnaires. Incomplete questionnaires and those who were not Malaysian citizens were excluded from the study.

### Sampling method

A complete list of universities in Malaysia was attained from the Ministry of Higher Education. Multistage cluster random sampling was employed to select universities. First, the universities were sorted into two groups - government and private universities. Second, the universities were further screened based on their ranking. To be selected for this study, the university must be listed under QS University Rankings Asia 2017/2018 for a government university and the Rating System for Malaysian Higher Education 2017 (SETARA) and Times Higher World University Rankings 2018 for a private university. Third, the universities that fulfilled the criteria were sorted according to their location (state). To note, Malaysian can be divided geographically into six zones. Zone A represents the Northern region, which consists of four states (Perlis, Kedah, Penang, Perak), Zone B represents the East coast region, which include of three states (Kelantan, Terengganu, Pahang), Zone C represents the Central region, which consists of another three states (Selangor, Federal Territories of Kuala Lumpur, Putrajaya), Zone D represents Southern region which consists of three states (Negeri Sembilan, Melaka, Johor), and finally, Zone E and Zone F which includes one state each (Sarawak and Sabah). Zones A, B, C and D are in Peninsular Malaysia, while Zones E and F are in East Malaysia.

**Fig. 1:**
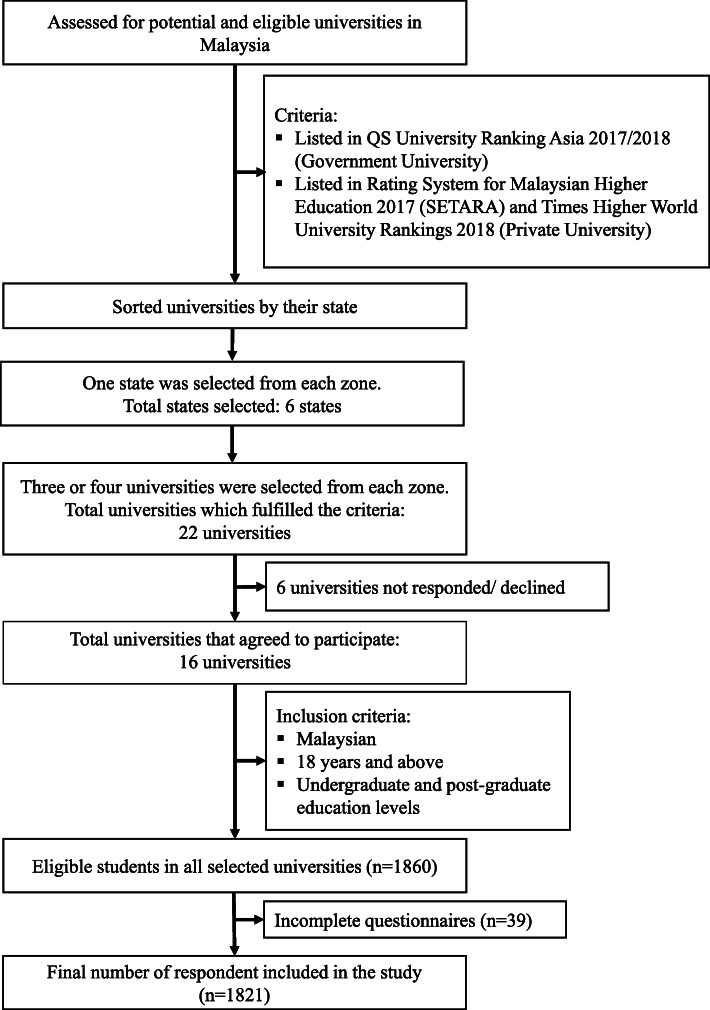
Flow chart of a multistage random sampling method. The list of universities in Malaysia was obtained from the Ministry of Higher Education

For this study, one state was chosen from each zone based on the lottery method (simple random sampling). We prepared six boxes to represent all the six zones. We then wrote the name of each state on the paper according to their zones and put them in the box that represents their zones. Later, we select the state by randomly chosen one paper from each box to represent the zone. Subsequently, from each state, using the similar lottery method mentioned previously, three or four universities that fulfilled the criteria were selected. Each selected university received an invitation letter and a brief explanation of the study. A confirmation letter was provided by the university which agreed to participate. There were twenty-two universities listed; however, only sixteen of them agreed to participate in this study. The schedule and participation of students in this study were arranged by the respective universities. Based on the sampling frame for each university, the eligible participants were selected via simple random sampling. On a respective day, data collection will be done by a group of trained researchers in the designated area (classroom, hall or hallway). The information sheet related to the study was distributed to the students, and they were briefed before completing the self-administered questionnaires. The written consent form from each student was taken before the data collection. A maximum of 45 min will be given to each student to answer the questionnaires, including the briefing time. During the session, body weight and height were measured to determine the current body mass index (BMI) of each student. The flow for the sampling method is shown in Fig. 1.

### Sample size

Calculation of sample size was done using the formula for testing the difference in proportions between two samples based on the previous study (CP West et al., 2011). To calculate the sample size, confidence level (95%), and values for power (80%) for both groups were desired. As this study followed the cluster sampling method, the sample size was multiplied by 1.2 for the design effect, making the total sample 1488. The final sample size is 1860 after taking consideration of 25% of possible dropouts.

### Instruments

The questionnaires were developed in dual languages, English and Bahasa Malaysia (the national language of Malaysia), and pre-tested among 80 university students who were not included in the study. A general information questionnaire was used to collect general demographic characteristics including age, gender, ethnicity, marital status, education level, the field of study, family monthly income, current living arrangements, smoking status, and alcohol consumption. A Cumulative Grade Point Average (CGPA) was used to evaluate the academic performance of the students. The CGPA determines the grades of the students for all semesters and courses that they had completed throughout the academic session [15]. Body mass index (BMI) was calculated from height and weight.

The WHO (2000) criteria were used to classify the BMI of the participants as underweight (< 18.5 kg/m^2^), average weight (18.5–24.9 kg/m^2^), overweight (25.0–29.9 kg/m^2^) and obesity (> 30.0 kg/m^2^). Psychosocial sections were developed by the researchers based on an extensive review of the literature [16–24]. The psychological part was assessed using the Generalized Anxiety Disorder-7 (GAD-7) questionnaire. GAD-7 is a 7-items of a self-report questionnaire used to screen the presence of anxiety and is often used in primary care and mental health settings. It measures the presence of symptoms of anxiety in the past 2 weeks of one’s daily life. The original version of the instrument was developed by Spitzer et al. [25]. It was subsequently validated in the Malay version by Sherina et al. [26]. It contains seven items which range from 0 (not at all) to 3 (nearly every day)., and cut-off scores are derived from 8 and above to shows the presence of anxiety,

### Ethical approval

Prior ethics approvals were obtained from the Ethics Committee for Research Involving Human Subjects, Universiti Putra Malaysia, Ministry of Higher Education Malaysia, and Ethics Committee in each selected university. Participation required written consent from each university and each student involved in this study.

### Data analysis

Data were entered into the Statistical Package for the Social Sciences (SPSS) software version 25, and the significance level was accepted as *p* < 0.05. Descriptive analysis (mean and standard division, frequency, and percentage) was conducted for all continuous and categorical data. The presence of anxiety among respondents was determined based on the cut of point value on GAD-7 (presence of anxiety GAD≥8 and absence of anxiety (GAD< 8). To determine the significant relationship between anxiety and variables, chi-square was used. All the variables with *p* < 0.05 on the chi-square were selected for further analysis. A multivariate logistic regression enter method was performed to determine the predictors of anxiety among students. To do multivariate logistic regression, coding was done on the dependent variable as 0 for the absence of anxiety and 1 for the presence of anxiety; also, the lowest prevalence group or sub-group from the categorical variable was taken as a reference category (RC).

## Results

### Descriptive statistics

Out of 1860 of total respondents, 1821 students participated in this study, giving a response rate of 97.9%. Among the participants, 1530 (84%) were aged between 18 and 22 years old, 271(14.9%) aged between 23 and 27 years old, and the rest, 20(1.1%) aged above 28 years old with 683 (37.5%) of them were male, and 1138 (62.5%) were female. The majority of the students were Malay (50.6%), and still single (98.8%). Most of the students were currently doing their bachelor’s degree (*n* = 1433, 78.7%), and in the first and second year of their study (*n* = 1120, 61.5%) A majority of the students were from Engineering, Manufacturing & Construction (*n* = 433, 23.8%), and Medicine (*n* = 362, 19.9%) courses. In this study, the highest percentage of CGPA was recorded between 3.0–3.74 (61.9%), with the lowest was between 2.0–2.24 (2.2%). From the data, the students mostly were from small middle-income families with a total of 1 to 5 members (63.2%), and a total income between RM 951 to RM3900 (39.6%). Three-quarters of the students (75.1%) were from urban areas, while almost two-thirds of them (60%) lived in their university college dormitories. Only a few students reported being involved with alcohol and smoking, 8.7 and 3.0%, respectively. All data are presented in Tables 1 and 2.

**Table 1:** Prevalence and relationship of anxiety based on the socio-demographic characteristics of participants (*n* = 1821)

Socio-demographic characteristic	Total number	Anxiety		Statistics
	N (%)	Yes (GAD ≥ 8) n (%)	No (GAD < 8) n (%)	
**Gender**				
Male	683	186(27.2%)	497(72.8%)	χ ^2^ = 1.75, df = 1, *p* = 0.08
Female	1138	343(30.1%)	795(69.9%)	
**Age group category**				
18–22	1530	449(29.3%)	1081(70.7%)	χ^2^ = 0.99 df = 2, *p* = 0.61
23–27	271	76(28.0%)	195(72.0%)	
> 28	20	4(20.0%)	16(80.0%)	
**Ethnicity**				
Malay	922	236(25.6%)	686(74.4%)	χ^2^ = 11.11, df = 3, *p* = 0.01*
Chinese	553	178(32.2%)	375(67.8%)	
Indian	165	53(32.1%)	112(67.9%)	
Others	181	62(34.3%)	119(65.7%)	
**Marital status**				
Single	1799	524(29.1%)	1275(70.9%)	χ^2^ = 0.98, df = 2, *p* = 0.61
Married	20	5(25.0%)	15(75.0%)	
Divorced & Widow	2	0(0.0%)	2(100.0%)	
**Monthly family income (RM)**				
RM950 and below	330	108(32.7%)	222(67.3%)	χ^2^ = 6.40, df = 3, *p* = 0.09
RM951-RM3,900	722	202(28.0%)	520(72.0%)	
RM3,901-RM8,400	504	132(26.2%)	372(73.8%)	
RM8,401 and above	265	87(32.8%)	178(67.2%)	
**Residency**				
Rural	454	113(24.9%)	341(75.1%)	χ^2^ = 5.07, df = 1, *p* = 0.02*
Urban	1367	416(30.4%)	951(69.6%)	
**Current smoking**				
Yes	54	22(40.7%)	32(59.3%)	χ^2^ = 3.96, df = 1, *p* = 0.04*
No	1767	507(28.7%)	1260(71.3%)	
**Alcohol consumption**				
Yes	158	73(46.2%)	85(53.8%)	χ^2^ = 24.69, df = 1, *p* < 0.01*
No	1663	456(27.4%)	1207(72.6%)	
**Poor sleep quality**				
Yes	779	328(42.1%)	451(57.9%)	χ^2^ = 112.57, df = 1, *p* < 0.01*
No	1042	201(19.3%)	841(80.7%)	
**Body Mass Index (BMI)**				
Underweight (Below 18.5)	290	104(35.9%)	186(64.1%)	χ^2^ = 10.95, df = 3, *p* = 0.01*
Normal (18.5–24.9)	1068	294(27.5%)	774(72.5%)	
Overweight (25.0–29.9)	318	82(25.8%)	236(74.2%)	
Obese (30.0 and above)	145	49(33.8%)	96(66.2%)	
**Number of people in household**				
1–5	1151	340(29.5%)	811(70.5%)	χ^2^ = 0.50, df = 2, *p* = 0.77
6–10	648	182(28.1%)	466(71.9%)	
≥ 11	22	7(31.8%)	15(68.2%)	
**Parents with tertiary education background**				
Yes	786	240(30.5%)	546(69.5%)	χ^2^ = 1.47, df = 1, *p* = 0.22
No	1035	289(27.9%)	746(72.1%)	

*Significant at *p* < 0.05;

**Table 2:** Prevalence and relationship of anxiety based on the academic characteristics of participants (*n* = 1821)

Academic characteristic	Total number	Anxiety		Statistics
	N (%)	Yes (GAD ≥ 8) n (%)	No (GAD < 8) n (%)	
**Level of education**				
Diploma	357	119(33.3%)	238(66.7%)	χ^2^ = 5.56, df = 3, *p* = 0.03
Degree	1433	403(28.1%)	1030(71.9%)	
Master	23	4(17.4%)	19(82.6%)	
Ph.D.	8	3(37.5%)	5(62.5%)	
**Academic year**				
1–2	1120	328(29.3%)	792(70.7%)	χ^2^ = 8.23, df = 2, *p* = 0.01*
3–4	630	191(30.3%)	439(69.7%)	
5 and above	71	10(14.1%)	61(85.9%)	
**Field of Study**				
Education	28	13(46.4%)	15(53.6%)	χ^2^ = 23.74, df = 6, *p* < 0.01*
Social Science, Business & Law	201	69(34.3%)	132(65.7%)	
Science, Mathematic &Computer	161	47(29.2%)	114(70.8%)	
Medicine	362	79(21.8%)	283(78.2%)	
Health Science	322	93(28.9%)	229(71.1%)	
Engineering & Manufacturing	433	116(26.8%)	317(73.2%)	
Others	314	112(35.7%)	202(64.3%)	
**CGPA** ^a^				
3.75–4.00	277	82(29.6%)	195(70.4%)	χ^2^ = 6.97, df = 3, *p* = 0.07
3.0–3.74	1128	323(28.6%)	805(71.4%)	
2.25–2.99	376	105(27.9%)	271(72.1%)	
2.0–2.24	40	19(47.5%)	21(52.5%)	
**Financial support for the study**				
Yes	998	252(25.3%)	746(74.7%)	χ^2^ = 15.46, df = 1, *p* < 0.01*
No	823	277(33.7%)	546(66.3%)	
**Current living arrangements**				
Parent’s home	439	142(32.3%)	297(67.7%)	χ^2^ = 10.08, df = 3, *p* = 0.01*
College Dormitory	1092	290(26.6%)	802(73.4%)	
Off-Campus	282	96(34.0%)	186(66.0%)	
Others	8	1(12.5%)	7(87.5%)	

*Significant at *p* < 0.05; ^a^Cumulative Grade Point Average

### Association between socio-demographic factors with anxiety

According to our study, the prevalence of students with the risk of anxiety was 29% based on the GAD-7 score more and equal to 8. Table 1 shows the relationship between socio-demographic factors and anxiety. Race (*p* = 0.01), residency (*p* = 0.02), smoking status (*p* = 0.04) and alcohol consumption (*p* = 0.00) were significantly associated with the risk of anxiety in bivariate analysis. The risk of anxiety in students with poor sleep quality was higher than students who had better sleep quality. BMI was also found to be statistically significant with the risk of anxiety (*p* = 0.01), where the percentage of obese and underweight students who had a risk of anxiety were slightly higher in these groups as compared to other BMI groups (GAD≥8).

### Association between academic characteristic with anxiety

Table 2 showed the prevalence and relationship of anxiety based on the academic characteristics of the students. Using bivariate analysis, academic year, the field of study, financial support for the study, and current living arrangement showed significant association with risk of anxiety. The percentage of diploma and PhD students who had a risk of anxiety (GAD≥8) were higher than other students. On the other hand, students in the first to the fourth academic year and students with the lowest CGPA (2.0–2.24) showed a higher prevalence risk of anxiety. Among all the courses, medicine, health sciences, and engineering & manufacturing exhibited a lower percentage of students with a risk of anxiety (GAD≥8). Besides that, the risk of anxiety was found to be higher in students with no financial support as compared to the students who received financial support. A similar trend was observed in students who lived with their parents and outside of the university.

### Association between psychosocial with anxiety

Based on the five psychosocial characteristic questions given to the students, all of them were statistically associated with anxiety at the bivariate level. As shown in Table 3, the risk of anxiety in students with a good friend(s) in the university and students who were actively involved in the society(s) were lower as compared to the students with no good friend(s) in the university and students who were not actively involved in the society(s). Conversely, based on the group of students who exhibited risk of anxiety (GAD> 8), students who doubted their future and students who were having problems with their friend(s) and lecturer(s) exhibited a higher percentage of having the risk of anxiety as compared to other groups.

**Table 3:** Prevalence and relationship of anxiety based on the psychosocial characteristics of participants (*n* = 1821)

Psychosocial characteristics	Total number	Anxiety		Statistics
	n (%)	Yes (GAD ≥ 8) n (%)	No (GAD < 8) n (%)	
**Having a good friend in university**				
Yes	1704	473(27.8%)	1231(72.2%)	χ^2^ = 21.47, df = 1, *p* < 0.01*
No	117	56(47.9%)	61(52.1%)	
**Having doubt regarding the future**				
Yes	1396	434(31.1%)	962(68.9%)	χ^2^ = 12.06, df = 1, *p* < 0.01*
No	425	95(22.4%)	330(77.6%)	
**Actively involved in a society**				
Yes	922	235(25.5%)	687(74.5%)	χ^2^ = 11.49, df = 1, *p* < 0.01*
No	899	294(32.7%)	605(67.3%)	
**Having problems with other students**				
Yes	385	160(41.6%)	225(58.4%)	χ^2^ = 37.06, df = 1, *p* < 0.01*
No	1436	369(25.7%)	1067(74.3%)	
**Having problems with any lecturer(s)**				
Yes	165	74(44.8%)	91(55.2%)	χ^2^ = 21.97, df = 1, *p* < 0.01*
No	1656	455(27.5%)	1201(72.5%)	

*Significant at *p* < 0.05;

### Predictors of anxiety

Multivariate logistic regression analysis was done to determine the predictor(s) of anxiety. The assumption of linearity, homoscedasticity, and normality of residuals were met, and the model was fit (χ2 = 246, df = 27, *p* = 0.00). Based on the analysis, out of fifteen variables significantly associated with the risk of anxiety in the bivariate analysis, ten of them were found to be the predictors of anxiety in our study. The strongest predictor of anxiety in our study was the academic year (Table 4). From the results, it showed that students who were in their first and second academic year exhibited a risk of anxiety 3.06 times more (OR = 3.06; 95% CI = 1.43–6.51) while students who were in their third and fourth academic year showed a risk of anxiety 2.95 times more (OR = 2.95; 95% CI = 1.35–6.47) as compared to those who were in year five and above of their study, respectively. On the other hand, the results showed that students who were doubting their future had a risk of anxiety 1.56 times more (OR = 1.56; 95% CI = 1.17–2.07) as compared to those who had no doubts about their future. The results also indicated that students who were not drinking alcohol (OR = 0.58; 95% CI = 0.39–0.85), students with good friend(s) in the university (OR = 0.44; 95% CI = 0.29–0.67), students who were actively involved in societies (OR = 0.64; 95% CI = 0.51–0.80), students with good relationships with other student(s) (OR = 0.59; 95% CI = 0.45–0.77) and lecturer(s) (OR = 0.64; 95% CI = 0.43–0.93), students with financial support (OR = 0.73; 95% CI = 0.58–0.92) and students with better sleeping quality (OR = 0.73; 95% CI = 0.58–0.92) were associated with lower prevalence risk of anxiety in this study.

**Table 4:** Predictors of anxiety based on multivariate logistic regression analysis

Variable	B	Wald	OR	***P***-value	95% CI
**Academic year**					
1–2	1.18	8.40	3.06	0.004	1.43–6.51
3–4	1.08	7.37	2.95	0.007	1.35–6.47
5 and above (Ref)					
**Financial support for the study**					
Yes	−0.31	7.12	0.73	0.008	0.58–0.92
No (Ref)					
**Alcohol consumption**					
Yes (Ref)					
No	−0.545	7.51	0.580	0.006	0.392–0.856
**Poor sleep quality**					
Yes (Ref)					
No	−1.01	80.99	0.36	0.00	0.29–0.45
**Body Mass Index (BMI)**					
Normal (18.5–24.9)	−0.47	5.31	0.62	0.02	0.41–0.93
Overweight (25.0–29.9)	−0.53	5.23	0.58	0.02	0.37–0.92
Obese (30.0 and above) (Ref)					
**Having a good friend(s) in university**					
Yes	−0.80	14.84	0.44	0.00	0.29–0.67
No (Ref)					
**Having doubt regarding your future**					
Yes					
No (Ref)	0.44	9.48	1.56	0.00	1.17–2.07
**Actively involved in societies**					
Yes					
No (Ref)	−0.44	14.76	0.64	0.00	0.51–0.80
**Having problems with other students**					
Yes (Ref)					
No	−0.51	14.77	0.59	0.00	0.45–0.77
**Having problems with any lecturer(s)**					
Yes (Ref)					
No	−0.44	5.27	0.64	0.02	0.43–0.93

*Significant at *p* < 0.05; Odd Ratio (OR); Confidence Interval (CI); Reference Group (Ref)

## Discussion

Anxiety is one of the risk factors in suicidal behavior, and it has been reported in many studies involving young people [27]. With the increase of mental health issues, suicidal thoughts and suicidal rates in many countries around the globe, it is becoming difficult to ignore all the factors that contribute to the problem. Studies have reported that teens and young adults are likely to be struggling with psychological distress and anxiety as compared to older adults [28]. Most of the university students were young adults ranging from age eighteen to twenty-four, where this is the age when the developmental stage transitions from late adolescence to adulthood [29, 30]. In this study, the prevalence risk of anxiety was recorded at 29%, where out of 1821 students, 529 of them had anxiety. Our data were similar to studies conducted at the Australian National University [17] and Yale University [31], with the prevalence risk of anxiety was at 17.5 and 29%, respectively. Nonetheless, higher anxiety was reported by Nour et al. (2016), where 62.4% of the students who participated in their study showed a potential risk of having anxiety, with 28.7% of them having clinically significant anxiety [32]. Similarly, a study conducted among Portuguese college students also depicted a higher prevalence of anxiety (32.8%) [33]. Based on the multivariate analysis, academic year, financial support for the study, alcohol consumption, poor sleep quality, body mass index (BMI), having a good friend(s) in the university, having doubt regarding the future, actively involved in the society, and having a problem with other students and lecturer(s) were found to be significantly associated and were the predictors for the risk of anxiety in our study.

The academic year showed the strongest association with risk of anxiety among university students in Malaysia. The significant relationship between anxiety and academic year can be contributed by several factors such as different approaches by each course, variation in assessment and grading system of each course and differences of teaching methods in the different academic years [18, 34]. A study has been reported that students in their early university years had difficulty adjusting to the new university life and having problem to handle everything independently [35]. Nevertheless, our data was inconsistent with a study conducted among engineering students in one of the public university in Malaysia, where, in their study, it showed that as the number of the academic year increased, the anxiety level among the students increased as the course becomes harder, and their workload increases [36]. The probable reason for the variation of data between studies can be due to factors such as sampling methods, sample size, the differences of the instrument used and the way the data was interpreted [37].

Malaysia is a multiracial country with different cultures, beliefs, and social activities [38]. In our study, drinking (alcohol) activity was one of the predictors for the risk of anxiety among university students in Malaysia. A study has shown that there is a positive correlation between drinking alcohol with anxiety [39]. Anxiety has been reported to be associated with frequent drinking and bingeing by anxious individuals to cope with their emotional distress [40]. As alcohol consumption is a common custom during social activities in many populations, the association between alcohol consumption with anxiety can be insignificant in some of the studies [41–43]. It was shown in a study conducted among Canadian Youth and university students in Hong Kong, where there was no significant relationship between drinking habit and alcohol consumption with anxiety were reported in these two studies [41, 44].

Good sleeping quality is important to the students during their study, as it is essential for their mental well-being and is interrelated with anxiety [45, 46]. In this study, we found that the sleeping habit was significantly associated with the risk of anxiety. Poor sleeping quality among university students is typical, especially during the examination period. Sleep deprivation may cause sleepiness, dizziness and impairment of cognitive and psychomotor of the students, which may lead to a decrease in concentration and difficulties in memorizing the subjects, thus affecting their academic performances [47]. Students who have sleeping deprivation tend to feel anxious, while those who have anxiety prompt to get trouble falling asleep at the same time. Our results were similar to studies conducted among university students in New Zealand, Egypt and Ethiopia [48–50].

The body mass index or BMI is widely known to have a positive correlation with anxiety. BMI is mainly influenced by dietary intake. Researchers have proved that there is an interrelation between emotional eating and psychological factors such as anxiety [51–53]. Previous work conducted among European university students indicated that eating behavior among university students could be influenced by many factors such as individual factors, social networks, the physical environment, and the macro-environment [54]. Emotional distress caused by factors such as examination and transition into adulthood can also affect the eating habit and cause them to eat more or less than the norm [55].

In this study, all the five-questions used to describe the psychosocial characteristics of the students were found to be the predictors of anxiety. A previous study showed that students with anxiety disorder tend to avoid all social interaction, isolate themselves, and hardly seek help from others [56]. They do not have good friends to share all their problems with and prefer not to engage in any social activities [57–59]. They tend to doubt their future, and, in some cases, they get into trouble with others [58]. All these findings are consistent with our current results.

### Strength and limitation

This study is the first cross-sectional study investigating the prevalence of anxiety among the government and private university students throughout Malaysia. As the questionnaires were conducted in dual language, it helped the students better understand and answer the questions accordingly. The large sample size in this study allows the researchers to perform more detailed and accurate statistical analysis. Nevertheless, several limitations of our study are noteworthy. First, since the questionnaire was conducted via self-report, the integrity of some data can be compromised as the students were able to discuss with their friends during the session and thus influenced their judgment when answering the questions. Secondly, data collection could not be done simultaneously. We took around 6 months to complete the data collection due to the lack of human resources, and more time was required to obtain approval from several universities. Because of this, some of the data collection was done right before or after the examination period, which may have an impact on the current mental health status of the students.

### Implication to practice

Stigma against a person with mental health issues prevents those with the problem from getting help. This study highlights the importance of studying the prevalence risk of anxiety and its predictors among university students in Malaysia. Given the limitations of the available data on the prevalence risk of anxiety, particularly among university students in Malaysia, the data obtained from this study is important for the development of specific interventions in reducing anxiety among university students.

## Conclusions

Among all the factors investigated in this study, only ten factors were the predictors for the risk of anxiety among university students in Malaysia. In summary, the prevalence risk of anxiety among the students is worrisome even though the prevalence rate is not as high as in other places. The universities and higher education bodies must place a much greater emphasis on mental health promotion to the students. Early screening and monitoring programs can be done to identify the high-risk students so that proper treatment can be given. These findings can be used to design appropriate and systematic interventions and programs to help students at risk of anxiety. Robust support and increase psychological assessment and monitoring among the students must be taken seriously to avoid higher prevalence rates in the future.

## Data Availability

Data and materials are available upon request from the corresponding author.

## Abbreviations

BMI: body mass index
GAD-7: Generalized Anxiety Disorder 7
PhD: Doctor of Philosophy
SPSS: Statistical Package for the Social Sciences
